# Facilitators and barriers to implementing provider-initiated HIV counselling and testing at the clinic-level in Ekurhuleni District, South Africa

**DOI:** 10.1186/s43058-022-00269-3

**Published:** 2022-02-15

**Authors:** Nolundi Mshweshwe-Pakela, Tonderai Mabuto, Nasiphi Ntombela, Mpho Hlongwane, Griffiths Kubeka, Deanna L. Kerrigan, Christopher J. Hoffmann

**Affiliations:** 1 The Aurum Institute, Johannesburg, South Africa; 2 The University of the Witwatersrand School of Public Health, Johannesburg, South Africa; 3 Department of Prevention and Community Health, George Washington University, Washington, DC, USA; 4 Department of Medicine, Johns Hopkins University School of Medicine, Baltimore, USA; 5 Department of Health, Behavior, and Society, Johns Hopkins Bloomberg School of Public Health, Baltimore, USA

**Keywords:** HIV testing, HIV testing services, Provider-initiated counselling and testing, Barriers and facilitators, Normalization

## Abstract

**Background:**

HIV testing is the entry point into the HIV care continuum and critical for HIV epidemic control. Facility-based HIV testing services (HTS) reach individuals who are already seeking clinical care and engaging with the medical care system. For this reason, individuals diagnosed with HIV during facility-based HIV testing are more likely to continue into HIV care. To increase the number of PLHIV who are diagnosed and initiated on ART, in 2015, the South African Department of Health instituted Provider-Initiated Counselling and Testing (PICT) policy—encouraging healthcare providers to recommend HIV testing, but this strategy remains under-utilized. We aimed to identify key constraints to the normalization of PICT implementation in 10 Ekurhuleni District healthcare facilities in South Africa.

**Methods:**

In-depth interviews were conducted with 40 healthcare workers (28 clinicians and 12 lay counsellors). Health care workers were purposefully selected to participate in the interviews, stratified by health facility and work category. Interviews were audio-recorded, transcribed, and translated for analysis. Thematic analysis was guided by the normalization process theory (NPT). NPT theory explains how practices are routinely embedded within organizational contexts. We used NVivo 10 software for qualitative data management.

**Results:**

Both clinicians and lay counsellors exhibited a clear understanding of the PICT policy— acknowledging its purpose and value. The identified barrier to normalization of PICT among clinicians was offering HIV testing based on suspicion of HIV despite understanding that PICT involves offering testing to all clients. Additionally, clinicians perceived PICT as incongruent with their clinical roles and perceived it to be lay counsellors’ responsibility. The main facilitator was the participation of all healthcare workers, specifically the presence of lay counsellors, although they also faced barriers such as a lack of workspace and under-appreciation.

**Conclusions:**

Use of NPT helped identify barriers that prevent the normalization of PITC and its integration into routine patient care. These barriers can be modified by low-cost interventions that promote congruence of PICT to the roles of clinicians and integrate the role of lay counsellors within the patient flow in the facility.

**Supplementary Information:**

The online version contains supplementary material available at 10.1186/s43058-022-00269-3.

Contributions to the literature

- Available literature shows that although PICT increases HIV testing, this policy is not fully implemented in healthcare facilities, especially by clinicians.
- For new interventions to be successfully implemented in a health care setting, they need to be incorporated to existing roles (normalized). Several factors affect this process and identifying such factors is a step towards mitigating identified barriers, leverage facilitators, and improve PICT delivery.
- These findings used NPT to highlight clinician barriers, role played by lay counsellors, and the potential role of full and collective participation of these healthcare worker categories to improve the delivery of PICT at the facility level.

## Introduction

HIV testing is the entry point into the HIV care continuum, which makes it an essential step for improved health outcomes for people living with HIV (PLHIV) and for epidemic control through antiretroviral treatment (ART) initiation and treatment as prevention. Facility-based HIV testing services (HTS) reach individuals who are already seeking clinical care and engaging with the medical care system [1, 2]. For this reason, individuals diagnosed with HIV during facility-based HIV testing are more likely to continue into HIV care compared to those diagnosed during community or mobile outreach [2, 3]. Facility-based HTS depends on providers recommending and, in some situations, performing HIV testing. To increase the number of PLHIV who are diagnosed and initiated on ART, in 2015 the South African Department of Health instituted a policy to encourage providers to recommend HIV testing [4, 5]. The policy refers to this approach as Provider Initiated HIV Counselling and Testing (PICT) which is described as follows: “PICT should be offered to all patients attending clinical services in both public and private sector. Health care providers should recommend HCT to all patients in a health facility, regardless of whether they show signs or symptoms of HIV infection” [6]. With the efforts to end the HIV epidemic, the UNAIDS proposed 90–90–90 targets to be achieved by the end of 2020: that 90% of people living with HIV (PLHIV) to know their HIV status, 90% of those to be initiated on ART, and 90% of those on ART to be virally suppressed [7]. With this, counsellors and clinics were given monthly testing targets and monthly reporting was requested from clinics, but there were no specific rewards or penalties for failing to meet prescribed targets [8]. Additionally, funders like the US President’s Emergency Plan for AIDS Relief (PEPFAR) offered funding to increase the number of people knowing their HIV status and linked to ART, working towards meeting the USAID targets. Despite the prioritization, prescribed testing targets, and monthly reporting, HIV testing continues to be offered to a small proportion of eligible patients [1, 8, 9].

Several approaches have been used to increase facility-based HTS [4, 10–12]. Several studies assessing facility-based PITC in low- and middle-income countries (LMICs) found a significant increase in HTS uptake when offered and provided by a healthcare provider compared to voluntary HIV testing or referral to onsite HIV testing [1, 10, 13]. Although a larger proportion of patients tested with PITC, the overall delivery of HTS remained low, reaching only a small portion of health facility attendees. Studies conducted mainly in developed countries and LMICs like USA, UK, Australia, France, Switzerland, Botswana, Zimbabwe, and Alabama reported that health care provider challenges leading to limited delivery of HTS within clinics include limited workspace; healthcare providers’ workload and PICT being viewed as adding to the workload, lack of confidence in counselling and HV testing skill, as well as dissatisfaction with salary [9, 14, 15]. These studies generally inductive analysis of in-depth interviews to describe specific challenges; interviews and analysis were not guided by implementation theory.

We sought to understand the normalization of provision of PICT in the health facility context. The process of normalization requires that health providers adopt the intervention and incorporate it into their routine work. The normalization process theory (NPT) is an explanatory model that describes the implementation of new interventions into health care facilities. The theory is organized into four constructs: (1) coherence, which refers to shared and personal beliefs of the purpose, value and demands of the practice; (2) cognitive participation, which refers to the commitment of actors to participate in the practice; (3) collective action, which refers to resources required to successfully perform HIV testing tasks, including working together in allocating and appropriately performing HTS tasks; and (4) reflexive monitoring, which refers to the level of reflection on, or appraisal of, the intervention by implementers, including whether it is likely to be perceived as advantageous for patients or staff [16]. Taken as a whole, these are processes through which interventions are integrated into routine health care practices and become normalized or part of the routine [17]. The ability to do this is affected by the health system and patient-related factors. These factors need to be clearly identified and characterized to inform the development of strategies that address them. Currently, there is limited literature describing factors that hinder normalization of PICT amongst healthcare providers, especially in low- and middle-income countries. Available literature has mainly examined individual, patients, and resource barriers to the successful delivery of HTS. We sought to understand key facilitators and barriers to the implementation of PICT in South African healthcare facilities using the theoretical framework of the NPT.

## Methods

### Study setting and data collection

Ekurhuleni is one of the five districts of Gauteng province of South Africa and the fourth largest Metropolitan municipality in South Africa. It is composed of urban and peri-urban residential areas with a total of 93 public health clinics and 6 public hospitals. The study was conducted in 10 public health facilities (6 primary health care centers (PHC), 3 community health care centers (CHC) and 1 district-level hospital) as part of a larger study to understand and increase PICT delivery [8]. These facilities were selected in coordination with the district-level Department of Health to achieve geographic diversity including distribution across North, South, and Eastern sub-districts. All the health facilities provided free HTS and HIV care and treatment services. HIV testing in South Africa includes the use of rapid point-of-care test kits and pre- and post-test counselling [6].

Between February and May 2017, we conducted in-depth interviews with 40 healthcare providers (medical doctors, professional nurses, and lay counsellors) who were involved with HIV testing. Table 1 summarizes the demographics of the interviewed healthcare providers. Three researchers developed an interview guide based on literature review, study objectives, and the NPT [17, 18] with the aim of understanding processes associated with provider-initiated facility-based HTS including challenges and facilitators to its optimal use and implementation. Participants were recruited using purposive sampling. In our purposive sampling approach, we aimed to document the views of the different providers which may have differing insights and experiences such as nurses, doctors, lay counsellors—depending on their availability/agree to participate in the interviews. We also aimed to get a representation of the 10 health facilities. Healthcare workers were approached at the health facility, given information about the study, and requested to participate. The researchers then secured appointments to discuss consent form information and to conduct the interview. Two experienced qualitative researchers were trained on using the interview guide and conducted the interviews. Both interviewers were females, one was a qualified Research Psychologist, and the other had a post graduate diploma in HIV/AIDS management. One interviewer was fluent in isiXhosa, isiZulu and English languages and the other was fluent in Sotho, Tswana and English languages. Both interviewers had experience in qualitative research design and in conducting in-depth interviews. Most of the interviews were conducted in isiXhosa, isiZulu, Sotho and Tswana with subsequent transcription and translation into English. Interviews were conducted in the health facilities, with only the interviewer and participant in the room and each interview lasted about 20–35 min. Saturation was monitored throughout the data collection process; researchers conducted several meetings to review and discuss audio-recordings of interviews soon after conducting them to identify recurring themes to decide whether to proceed or stop conducting additional interviews.

**Table 1:** Participants characteristics (*N*=40)

Demographic characteristics	***N***	%
Gender		
Male	6	15
Female	34	85
Professional status		
Medical doctors	2	5
Professional nurses	26	65
Lay counsellors	12	30
Experience in current HTS role (years)		
<1–5	20	50
5–10	16	40
11–15	2	5
> 15	2	5

### Data analysis

A qualitative data analysis software program, Nvivo 10, was used for data management. NPT was used to develop the codebook and organize codes. We analysed the transcripts using thematic analysis. All categories constituting NPT constructs were assigned a code domain and specific subcodes were developed for each of the 4 domains. Any themes representing the 4 NPT domains were extracted and assigned to the specific codes. One researcher developed a first draft codebook and then discussed it with a senior researcher and agreed on themes and codes to be included. Two researchers then selected 5 transcripts and used the codebook to concurrently analyse them. The two researchers discussed the codes, agreed on final codes and themes and updated the codebook. Transcripts were then divided equally amongst the 2 researchers for the rest of the analysis.

## Results

We conducted 40 in-depth interviews with healthcare providers: 26 professional nurses; 2 medical doctors, and 12 lay counsellors (Table 1). The majority (34) were women and the median years of work experience in the current role were 5.5 years (interquartile range, IQR, 2, 7).

### Coherence

Health care workers views of PICT and the universal PICT policy demonstrated their understanding of the purpose of these processes with the broader workplace goals of improving patient health and care outcomes. Healthcare providers correctly described the Department of Health PICT policy (offering HIV testing to all patients): “I think provider-initiated testing counselling says that any patient that comes, I offer them HIV [testing] whether they came with headache or having whatever…” (*H-010-02, nurse*). Many healthcare providers also articulated a justification explaining that you cannot tell whether a person is HIV positive just by looking at them. This highlighted the importance of universal testing as described in national PICT policy. This was described by one provider as follows: “It’s usually each and everybody, we do not say you will see this one thin. You will see this one big, but still they might have HIV. So we offer each and everybody” (S-003-01, *nurse*) (Table 2).

**Table 2:** Facilitators and challenges to normalization of HTS

Facilitator	Quotes	Challenge	Quotes
Coherence			
Health care workers’ understanding of policy to test all patients.	“I think provider-initiated counselling and testing says that any patient that comes, I offer them HIV [testing] whether they came with headache or having whatever…” (H-010-02, nurse) “It’s usually each and everybody, we do not say you will see this one thin. You will see this one big, but still they might have HIV. So we offer each and everybody” (S-003-01, nurse).	Targeted testing	“By their clinical pictures, physical things…another one maybe by the signs/symptoms that they are mentioning …especially when they have STIs, I encourage them to go.” (E-002-02, nurse) Well from our wing it’s mostly initiated based on symptom, so 99% of patients who come in will have features of something that could possibly be immunodeficiency then we’d like to test you for HIV”. (H-010-04, doctor)
		Lay counsellors relied on clinicians to refer patients	“If I’m going to leave my room and go promote when my line gets longer here, it means I’m still busy there and I am wasting time. And if only health promoters would promote it every day and other sister-professional nurses would promote it.” (E-002-01, counsellor)
**Cognitive participation**			
Lay counsellors understood HTS as their duty and were committed to providing it.	“I know how to do my work [HIV counselling and testing]. Patients sometimes don’t want counselling, but I tell them that I have to do everything accordingly…” (N-001-0, counsellor)	Clinicians (doctors & nurses) felt that HTS was not their work.	“I think doctors & nurses’ involvement would be a good thing but due to the workload we are not able to do it personally. We have a lot of patients who are waiting for us and we do have counsellors who are employed to do the HIV testing…” (E-003-02, nurse) “We don’t keep patients who have come to test to ourselves because we have work to do. The counsellors are there for testing and if we take the patients they will have nothing to do”. (E-003-02)
**Collective action**			
Division of roles	“As I told you, we do assess patients and then we offer [HTS], if the patient agrees we send them to the counsellors, and they test and counsel them.” (S-001-01, nurse)	Clinicians not providing HTS even when lay counsellors are nor around	“We knock off at 2 and you find that people come to the clinic to test knowing that the clinic closes at 4 pm. When they get here they are told that the counsellors have left…” (N-003-01, counsellor))
		Low compensation of counsellors for the work.	“Thing is we counsellors in the hospital we are called “volunteers”, so you can’t cover a volunteer. We are doing this because … I love what I am doing but I won’t stay in this profession for long, I am still looking for greener pastures”. (H-001-01, counsellor)
		Limited HTS workspace	“We always have test kits. Every Monday the nurse orders test kits for us. We have everything except the working space.” (N-001-01, counsellor) We don’t have enough working spaces. We only have 1 room to test the patients and there is always a queue that side”. (E-002-02, nurse)
		Long queues and huge work load	Remember you can get 5 or 10 patients at a time, isn’t it? Now there’s 1 counsellor, they can only see 1 patient at a time, then the patients get irritated” (H-010-02, doctor)
**Reflexive monitoring**			
Emphasis on need to test all patients to prevent stigma and discrimination	10-1: It is easier to get people to agree when they see that everyone is going, it is not just me who’s being picked saying no you must come and also this picking…obviously it’s stigmatizing so the person will wonder, why me, (Hospital doctor).	HTS lower priority compared to patient’s reason for visiting clinic	“You keep thinking that now if this patient goes there he’s going to spend 15 minutes then maybe I’ll also send him to X-ray that’s another 15-30…then you say no no….this one [HIV testing] can wait.” (H-010-02, doctor)

Despite the understanding of, and stated agreement with, the PICT policy and policy justification, there was a lack of coherence with implementation. The lack of coherence was illustrated by the same clinicians reporting actual practices of offering HIV testing based on suspicion of HIV. Specifically, the presence of chronic illness, wasting, or sexually transmitted diseases was what prompted most clinicians to recommend testing.

By their clinical pictures, physical things…another one maybe by the signs/symptoms that they are mentioning …especially when they have STIs, I encourage them to go [for an HIV test]. (E-002-02, nurse)

Well from our wing it’s mostly initiated based on symptom, so 99% of patients who come in will have features of something that could possibly be immunodeficiency then we’d like to test you for HIV. (H-010-04, doctor)

Lay counsellors also expressed a clear understanding that all patients attending the clinic should be offered an HIV test. They however mainly tested patients referred by the clinicians. They were able to offer PICT to patients when they had no patients queuing for HIV testing. One lay counsellor narrated as follows:

If I’m going to leave my room and go promote when my line gets longer here, it means I’m still busy there and I am wasting time. And if only health promoters would promote it every day and other sister-professional nurses would promote it. (E-002-01, counsellor)

### Cognitive participation

The level of commitment to PICT  differed between clinicians (doctors and nurses) and counsellors. Clinicians stated that they did not see it as their duty to perform HTS. In addition, several nurses stated that they lacked the counselling skills needed to engage patients with HTS. Clinicians (both doctors & nurses) stated they were comfortable with recommending HIV testing, but not with being involved in providing the actual test. In contrast, HTS counsellors described full participation in the delivery of PICT.

I think doctors & nurses’ involvement would be a good thing but due to the workload we are not able to do it personally. We have a lot of patients who are waiting for us and we do have counsellors who are employed to do the HIV testing… (E-003-02, nurse) (Table 2).

I know how to do my work [HIV counselling and testing]. Patients sometimes don’t want counselling, but I tell them that I have to do everything accordingly… (N-001-0, counsellor)

### Collective action

There was a common practice of role division amongst healthcare providers in the provision of PICT. HIV testing was mainly recommended by the clinicians for delivery by lay counsellors. While this approach allowed for a degree of skill specialization, HTS services broke down when counsellors were not available due to the limited working hours of lay counsellors in the facility. When lay counsellors were unavailable, HTS was not provided. This suggested a lack of full engagement in collective action.

As I told you, we do assess patients and then we offer [HTS], if the patient agrees we send them to the counsellors and they test and counsel them. (S-001-01, nurse)

We knock off at 2 and you find that people come to the clinic to test knowing that the clinic closes at 4 pm. When they get here they are told that the counsellors have left… (N-003-01, counsellor)

An additional barrier to a team-based approach was the substantial difference in status of the nurses and doctors compared to lay counsellors. Lay counsellors received small stipends, limited training, and were not embraced as part of the clinical team, often being left out of facility meetings including those regarding HTS: “If I were to tell you… actually they don’t value us, they don’t count us. We don’t have a say that is why we end up not knowing where we stand, because we don’t have a say” (E-001-01, counsellor*).*

We counsellors in the hospital we are called “volunteers”, so you can’t cover [no employee benefits] a volunteer. We are doing this because I love what I am doing but I won’t stay in this profession for long, I am still looking for greener pastures. (H-001-01, counsellor)

This further challenged creating a team dedicated to a collective goal.

Lay counsellors also described lacking the needed private space to provide HTS– suggesting lack of full support from clinic management.

We always have test kits. Every Monday the nurse orders test kits for us. We have everything except the working space. (N-001-01, counsellor)

We don’t have enough working spaces. We only have one room to test the patients and there is always a queue that side. (E-002-02, nurse) (Table 2).

### Reflexive monitoring

Some health workers emphasized the value of offering HIV testing to all clients, suggesting it should be considered similar to checking vital signs. One clinician further mentioned that offering an HIV test to everyone may reduce the stigma of HIV testing.

It is easier to get people to agree when they see that everyone is going, it is not just me who’s being picked saying no you must come and also this picking…obviously it’s stigmatizing so the person will wonder, why me. (H-010-08, doctor).

However, when compared to other practices embedded in routine practice, HIV testing was perceived to have challenges that made it a lower priority. A doctor narrated this as follows: “You keep thinking that now if this patient goes there he’s going to spend 15 minutes then maybe I’ll also send him to X-ray that’s another 15-30…then you say no no…. this one [HIV testing] can wait” (H-010-02, doctor). This illustrates the gap in reflexive monitoring, that despite identifying gaps in delivery, clinicians were not motivated to change the current approach to PICT.

## Discussion

We used the NPT to frame and describe normalization of PICT within the public health care setting in South Africa. Interviews with doctors, nurses, and lay counsellors provided insight into beliefs that can support PICT delivery as well as current constraints. The barriers to PICT normalization were related to coherence and collective action. This study showed that while nearly all healthcare providers embraced the concept of universal delivery of PICT, a lack of coherence existed between the concept and actual practice. Facilitators to normalization of PICT were related to cognitive participation - availability of trained lay counsellors and staff’s awareness of and willingness to support the policy.

Prior work on PICT delivery has identified workload, staff shortage, and long queues as barriers to implementing PICT [19–21]. Most studies have not sought to understand the overall acceptance or normalization of PICT by various cadres of health care workers nor have they generally used a theoretical framework. One study that also used NPT, evaluated the introduction of PICT into clinics in Cape Town, South Africa. This study was conducted in the setting of a clinical trial [21]. The authors reported similar findings of the general coherence of PICT with the overall health care goals. Also similar to our findings, the authors reported a degree of resistance from some nurse clinicians to provide PICT. In the short term, some of the resistance was overcome by PICT champions. Our study builds on this early implementation study in suggesting that over time and without the infrastructure of a clinical trial, coherence with the intervention may decrease. If this occurs, intervention normalization may fail to occur and implementation may diminish over time [22].

A systematic review with studies mainly conducted in developed countries reported that nurses’ views towards PICT were affected by the extent to which they perceived it to enhance or damage care. In some cases, it was affected by whether they saw it as part of their professional role. Nurses often cited staff high patient volumes, staff shortages and structural problems as barriers to offering and providing PICT. All these suggest that nurses did not perceive PICT as their role, findings which were similar to the current study [23].

We observed some discordance between which patients should be offered PICT and which patients some health care workers offered PICT to. Finally, a lack of role concordance between the primary responsibilities of the clinicians and the tasks they were asked to do as part of PICT led to potential under-delivery of PICT and a breakdown in collective action. These areas suggest two approaches may be needed to increase PICT delivery: (1) management support or champions to promote a uniform message of which patients should receive PICT and (2) a reconsideration of the specific roles of clinicians (nurses and doctors) in PICT to assure that roles fit within overall job activities rather than feeling like added activity with a different skill set.

Strengths of the study include the use NPT to frame an evaluation of PICT delivery in a real-world setting and conducting the study in multiple public clinics offering routine services. Limitations of this study include conducting interviews in several languages which may have limited both interviewers’ comfort in phrasing and explaining the questions and participants’ comfort in expressing their responses. As a qualitative study, we used purposive sampling to select our participants. We however attempted to achieve representation across the10 health facilities and different types of healthcare workers. Generalizability of findings may be limited to Ekurhuleni district and similar contexts.

## Conclusions

We found a wide understanding of the purpose of PICT and articulation of the value of PICT, but a lack of coherence and collective action with implementation. This likely contributes to the challenges with increasing clinic-based HIV testing. Implementation research strategies can be used to explore context-specific barriers to normalization and adapt existing interventions to address such barriers. These can include low-cost interventions that integrate PICT to existing clinic flow, e.g., offer and provide PICT at waiting area; strengthening skills and motivation of clinicians to offer and provide PICT.

## Supplementary Information

**Additional file 1.**

## Data Availability

The datasets used and/analysed for this study are available from the corresponding author on reasonable request
