# Supplementary material for: Facilitators and barriers to implementing provider-initiated HIV counselling and testing at the clinic-level in Ekurhuleni District, South Africa
Source: Implement Sci Commun. 2022 Feb 15;3:19. doi: 10.1186/s43058-022-00269-3 (PMC8845300; doi:10.1186/s43058-022-00269-3)
Supplement: Supplementary file 1 — Additional file 1. [file 43058_2022_269_MOESM1_ESM.docx]

**Introduction**

Thank you for agreeing to be part of the interview. The aim of this interview is to get an understanding of your own perspectives and experiences with HIV testing services within your facility.

1. **Participant introductions**
   1. Tell me about your work role in this facility facility.
   2. Tell me about your background (education/previous work) and your experience working within the facility
2. **Main questions**
3. The South African Provider Initiated Counselling and Testing policy, like any other policy, is interpreted in different ways by people. What is your own understanding of provider initiated testing and counselling and the purpose it sets out to achieve?
4. The PICT guidelines do not prescribe/specify how PITC should be delivered. As a result, different facilities implement PITC using various approaches. Please talk about how provider-initiated counselling and testing is implemented in this facility?
   1. For you specifically, how has PITC been incorporated into your roles and responsibilities?
   2. How do you go about the process of identifying/selecting patients for PITC e.g. do you look at particular symptoms.
5. Can you tell me about your experiences with providing PITC in this facility?
   1. Facilitators to providing the service.
   2. Challenges encountered with providing the service.

Let’s talk about some of your thoughts around PITC

1. What are your personal thoughts about the participation of doctors or nurses in delivering HIV testing and counselling within healthcare facilities?
2. The success of PITC has been generally minimal. From your own experiences, what do you think are the reasons for this?
3. Based on the discussion we have just had, what are your thoughts on how PITC should be taken forward?

**END OF INTERVIEW**
